# Speech perception consistency facilitates initial lexical activation, but not speech perception flexibility

**DOI:** 10.1038/s41598-026-47943-3

**Published:** 2026-04-09

**Authors:** Brian W. L. Wong, Arthur G. Samuel, Efthymia C. Kapnoula

**Affiliations:** 1 BCBL, Basque Center on Cognition, Brain and Language, Donostia-San Sebastian, Spain; 2 University of the Basque Country (UPV-EHU), Donostia-San Sebastian, Spain; 3 Department of Psychology, Stony Brook University, New York, USA; 4 Ikerbasque, Basque Foundation for Science, Bilbao, Spain

**Keywords:** Speech perception, Consistency, Individual differences, Lexical activation, Neuroscience, Psychology, Psychology

## Abstract

Speech perception consistency refers to the similarity between a listener’s responses to repeated presentations of the same speech sound (e.g., along a /ba/–/pa/ continuum). Although research has demonstrated multiple advantages of consistency, its role in initial lexical activation (i.e., early activation of word candidates) and speech perception flexibility (e.g., the ability to recover from misleading speech input) has not been tested. We investigated the role of consistency in spoken-word recognition among Spanish (L1)–English (L2) bilingual listeners. Focusing on the bilabial stop contrast (/b/–/p/), consistency was measured in a task where participants repeatedly rated speech sounds on a continuous scale (Visual Analog Scale), whereas initial lexical activation and speech perception flexibility were assessed using a word-to-picture matching task with eye-tracking (Visual World Paradigm). Seventy Spanish–English bilinguals completed these tasks in both languages. Listeners with higher consistency exhibited stronger initial lexical activation for acoustically compatible word candidates across /b/ and /p/ in L1 Spanish and for /p/ in L2 English. However, consistency was not associated with speech perception flexibility. These findings suggest that speech perception consistency primarily supports early lexical activation during spoken-word recognition, while playing a more limited role in later processes involved in revising initial misinterpretations.

**Supplementary Information:**

The online version contains supplementary material available at 10.1038/s41598-026-47943-3.

## Introduction

Speech perception is a remarkable feat of the human mind. It requires rapid and efficient mechanisms to extract linguistic meaning from a continuously changing input. From such a rapidly changing acoustic waveform, listeners are able to extract linguistic structure, recognize words and sentences, and understand each other effortlessly in everyday communication. Yet beneath this apparent ease lies an extraordinary computational challenge: the speech signal is highly variable, influenced by talker differences, speaking rate, and contextual factors^1–4^.

To overcome this variability, the perceptual system must strike a delicate balance between stability and flexibility^5,6^. Stability ensures consistent mapping from variable acoustics to linguistic categories, while flexibility allows listeners to adapt to variability in the signal^7,8^. Listeners differ in the extent to which they exhibit these two properties, with this balance reflected in two key constructs at the subphonemic level (i.e., below the level of phoneme categories): (1) consistency, defined as the stability of listeners’ perception of the same stimulus (reflected in the consistency of their responses to repeated presentations of the same stimulus), and (2) gradiency, defined as sensitivity to within-category variation (fine-grained acoustic differences among sounds mapped to the same phoneme). Speech perception consistency has only recently begun to receive attention and has not yet been integrated into current models of speech processing^9,10^, leaving its functional roles and origins largely unexplored. In the present study, we focus on two potential functions of consistency: initial lexical activation and speech perception flexibility.

Consistency can be measured reliably using a Visual Analog Scale (VAS), in which participants hear multiple instances of each sound from a test continuum, and respond by clicking at any point along a continuous line to indicate what they heard^11,12^. For example, the sounds can come from a continuum between /b/ and /p/, constructed by manipulating voice onset time (VOT). VOT is the temporal interval between the release of a stop consonant and the onset of vocal fold vibration. It serves as the primary acoustic cue distinguishing voiced and voiceless stop consonants in many languages, including Spanish and English, which are the primary languages of interest in the present study. By examining the stability of participants’ responses across repeated presentations of the same stimulus, we can quantify their speech perception consistency.

**Fig. 1:**
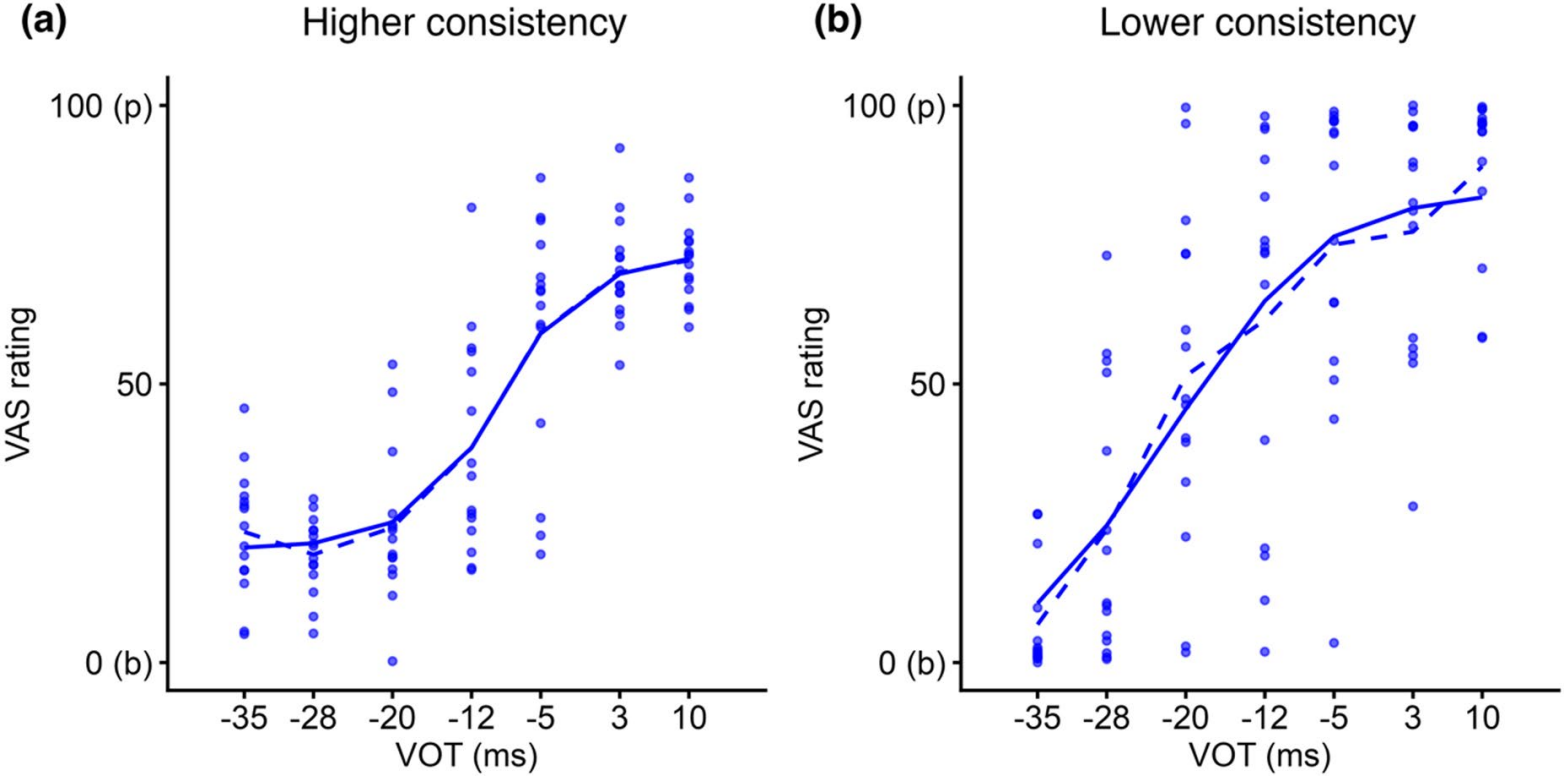
Examples of Spanish VAS data showing different consistency of two participants. Each dot represents a participant’s response on one trial. The solid line is the fitted curve based on the rotated logistic function, while the dashed line connects the average response at each VOT value for that participant. Participant (**a**) shows higher consistency across repeated presentations of the same stimulus, as indicated by the clustering of dots around the fitted curve, whereas participant (**b**) shows lower consistency, as indicated by the more dispersed dots around the fitted curve.

Individual differences in consistency have been reported in previous studies^11,13–15^. These differences are illustrated in Fig. 1, which shows Spanish VAS data from two participants in the current study. Each dot represents a participant’s response on an individual trial. Tighter clustering of dots (vertically) around the fitted curve at the same VOT value indicates higher consistency (left panel), whereas greater dispersion of dots reflects lower consistency (right panel).

A growing body of research has begun to investigate the functions of speech perception consistency in spoken language comprehension, as well as in broader language and reading abilities. This work suggests that consistency plays an important role across these domains. Specifically, higher consistency has been positively associated with reading and language skills^12,16–18^, improved learning of novel sound contrasts^14,15,18^, better speech-in-noise perception^19,20^, more adaptive use of acoustic cues across phonological contrasts^21^, and more accurate perception of novel accents^22^. Collectively, these findings indicate that listeners with higher consistency tend to perform better across multiple domains of speech perception and language learning.

Despite these advantages, the potential role of consistency in shaping initial lexical activation and speech perception flexibility remains unclear. The present study addresses this gap by examining how individual differences in consistency relate to both initial lexical activation and speech perception flexibility. The data analyzed here were collected as part of a larger study, another part of which examined the role of gradiency in spoken-word recognition^23^. The present analyses were guided by a priori hypotheses concerning consistency and address a distinct theoretical question that was not examined in the prior report. Analyses that were not hypothesis-driven are explicitly labeled as exploratory.

In the present experiment, Spanish–English bilinguals completed a VAS task assessing consistency. They also completed a Visual World Paradigm (VWP^24,25^) task, in which they listened to spoken words while viewing pictures on a screen, and their eye movements to the pictures were recorded in real time. Participants heard items such as ϸeachball, in which the initial consonant varied along a continuum between /b/ and /p/ (with [ϸ] representing any token along this continuum). Initial lexical activation was operationalized as early looks to /b/- and/p/-onset referents (i.e., visual objects corresponding to the spoken words; e.g., *beachball* and *peachpit*) as a function of VOT. Specifically, for this measure, we only included fixations occurring before the point of disambiguation (POD), defined as the point in the auditory signal at which the input uniquely specifies the target word (e.g., the transition from *beach* to *ball* in *beachball*). Increased looks to the /b/-onset referents when VOT was low and increased looks to the /p/-onset referents when VOT was high were taken to indicate stronger initial lexical activation.

As a result of this manipulation, the VWP task exposed listeners to spoken “garden-path” words designed to mislead participants and, as such, allowed us to measure speech perception flexibility following an initial misinterpretation. In a garden-path utterance, words initially match one lexical candidate but later unfold into a different word. Such stimuli allow us to observe how listeners recover from ambiguous or misleading information as speech unfolds over time. Upon hearing *peachball*, listeners were initially likely to fixate on *peachpit* (the “competitor”). As later acoustic input was consistent with *beachball* (“the target”), listeners shifted their gaze to the target image, signaling recovery from the garden-path. The rate and latency of gaze shifts toward the target reflect speech perception flexibility. Thus, the VWP provides a measure of both initial lexical activation and speech perception flexibility.

**Fig. 2:**
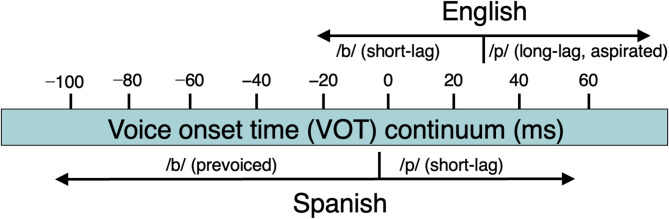
VOT differences between English and Spanish/b/–/p/contrasts. Adapted from^28,29^.

The present study focuses on the voicing contrast /b/–/p/ in Spanish and English. This comparison is especially informative because VOT, the primary acoustic cue distinguishing these sounds, differs substantially between the two languages. Spanish typically exhibits VOTs of approximately −80 ms for /b/and 16 ms for /p/^26^, whereas English /b/ and /p/ average around 10 ms and 65 ms, respectively^1,27^(see Fig. 2). In Spanish, /b/ is prevoiced, meaning that vocal fold vibration begins before the consonant is released, yielding a negative VOT, whereas Spanish /p/ is acoustically closer to English /b/.

Our main research questions concern whether speech perception consistency predicts initial lexical activation and speech perception flexibility. We predicted that listeners who exhibit lower speech perception consistency would show reduced initial lexical activation of acoustically compatible words. Specifically, they would look less to the /b/-onset referent when VOT was low and to the /p/-onset referent when VOT was high, compared to listeners with higher consistency. This prediction is grounded in previous findings showing that lexical activation is delayed when the input is degraded or noisy (e.g., cochlear implant users and normal-hearing listeners presented with noise-vocoded speech^30–32^). Listeners with lower consistency may experience reduced stability in the mapping between acoustic cues and speech categories, leading to increased uncertainty during speech processing. This uncertainty may induce a “wait-and-see” strategy in the early stages of spoken-word recognition, whereby initial activation of lexical candidates is delayed to avoid premature commitment until additional information becomes available^33^. Based on this reasoning, we predicted that the relationship between consistency and initial lexical activation would be present in both the first language (L1) Spanish and the second language (L2) English, as increased perceptual uncertainty is expected to influence lexical processing regardless of language. In contrast, we did not expect consistency to play a major role in speech perception flexibility in either L1 Spanish or L2 English, because flexibility reflects the ability to recover from misleading information, which is less directly related to the stability of the mapping between acoustic cues and phoneme categories.

## Results

### Preliminary Analyses

**Fig. 3:**
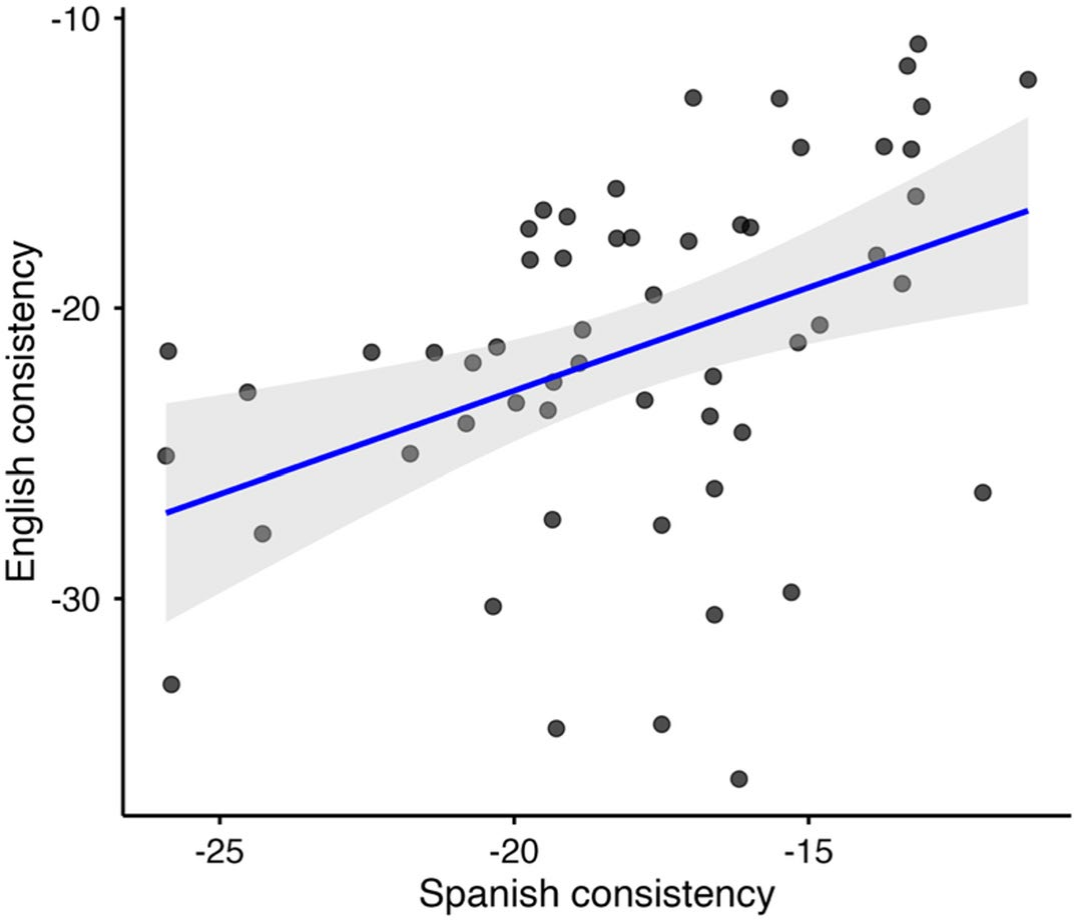
Correlation between L1 Spanish consistency and L2 English consistency. The blue line indicates the fitted least-squares regression line, and the grey shaded area represents its 95% confidence interval. Each dot indicates data from one participant. Adapted from^23^.

As was previously illustrated in Fig. 1, participants varied in their degree of consistency as measured by the VAS task. As reported in the Supplementary Materials of^23^, consistency showed a positive correlation across Spanish and English (*N* = 56), Spearman’s rho (*ρ*) = 0.44, *BF* = 27.18, providing strong evidence that individuals who were more consistent in one language also tended to be more consistent in the other language. This relationship is visualized in Fig. 3.

## Main analyses

### Does consistency predict initial lexical activation?

Our first research question examined whether consistency predicts initial lexical activation in L1 Spanish and L2 English. Initial lexical activation was indexed by participants’ fixations to /b/- and /p/-onset pictures as a function of VOT prior to the POD. Accordingly, this measure captures early lexical activation based on phoneme onset, independent of word offset timing or participants’ final responses. Given that lower VOT values correspond to /b/ and higher VOT values correspond to /p/ in both Spanish and English, greater proportions of looks to /b/-onset pictures at lower VOT values and greater proportions of looks to /p/-onset pictures at higher VOT values were taken to indicate stronger initial lexical activation of acoustically compatible words across languages. This was tested by the consistency × VOT interaction (rather than the main effect of consistency), which aligns with our hypothesis that consistency reflects more stable cue-to-category mapping. This approach allows us to assess the effect of consistency on lexical activation independently of potential differences in overall looking patterns or other biases. Full statistical details and model specifications are reported in the *Statistical Analyses* section of the Methods.

**Fig. 4:**
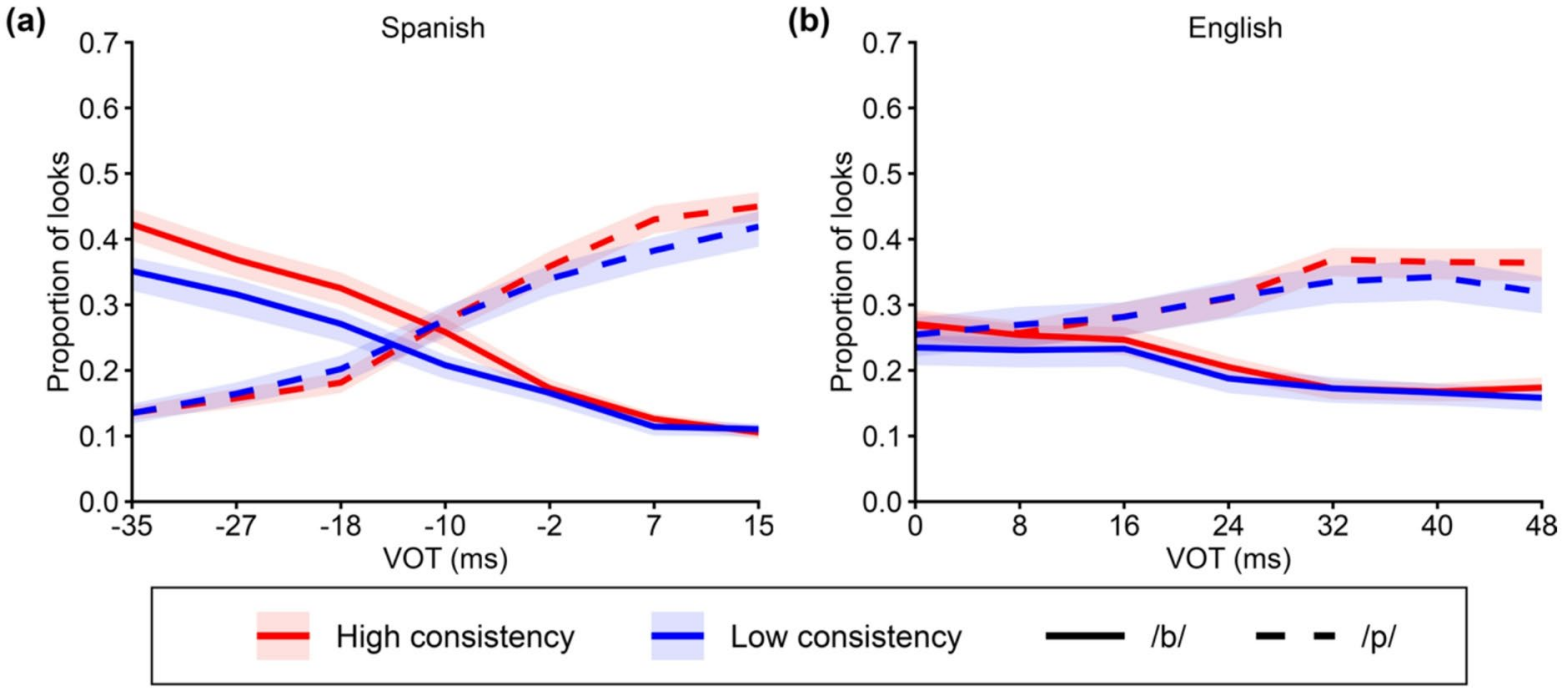
Proportion of looks to /b/-onset pictures and /p/-onset pictures as a function of VOT and consistency in **(a)** Spanish and **(b)** English. “Low consistency” and “high consistency” were defined by a median split. “/b/” and “/p/” refer to fixations on pictures whose target words began with /b/ and /p/ before the point of disambiguation (POD), respectively. Shaded ribbons indicate ± 1 standard error of the mean (SE) around each curve, computed across participants at each VOT.

Figure 4a and b illustrate how the proportion of looks to /b/-onset and /p/-onset pictures prior to the POD varied as a function of VOT and consistency in Spanish (*N* = 65) and English (*N* = 52), respectively. As expected, listeners made fewer looks to /b/-onset pictures and more looks to /p/-onset pictures as VOT increased. Crucially, listeners with higher consistency exhibited stronger VOT-compatible looking patterns, showing more looks to /b/-onset pictures at lower VOT values and more looks to /p/-onset pictures at higher VOT values. This pattern was confirmed by the statistical analyses reported below.

**Fig. 5:**
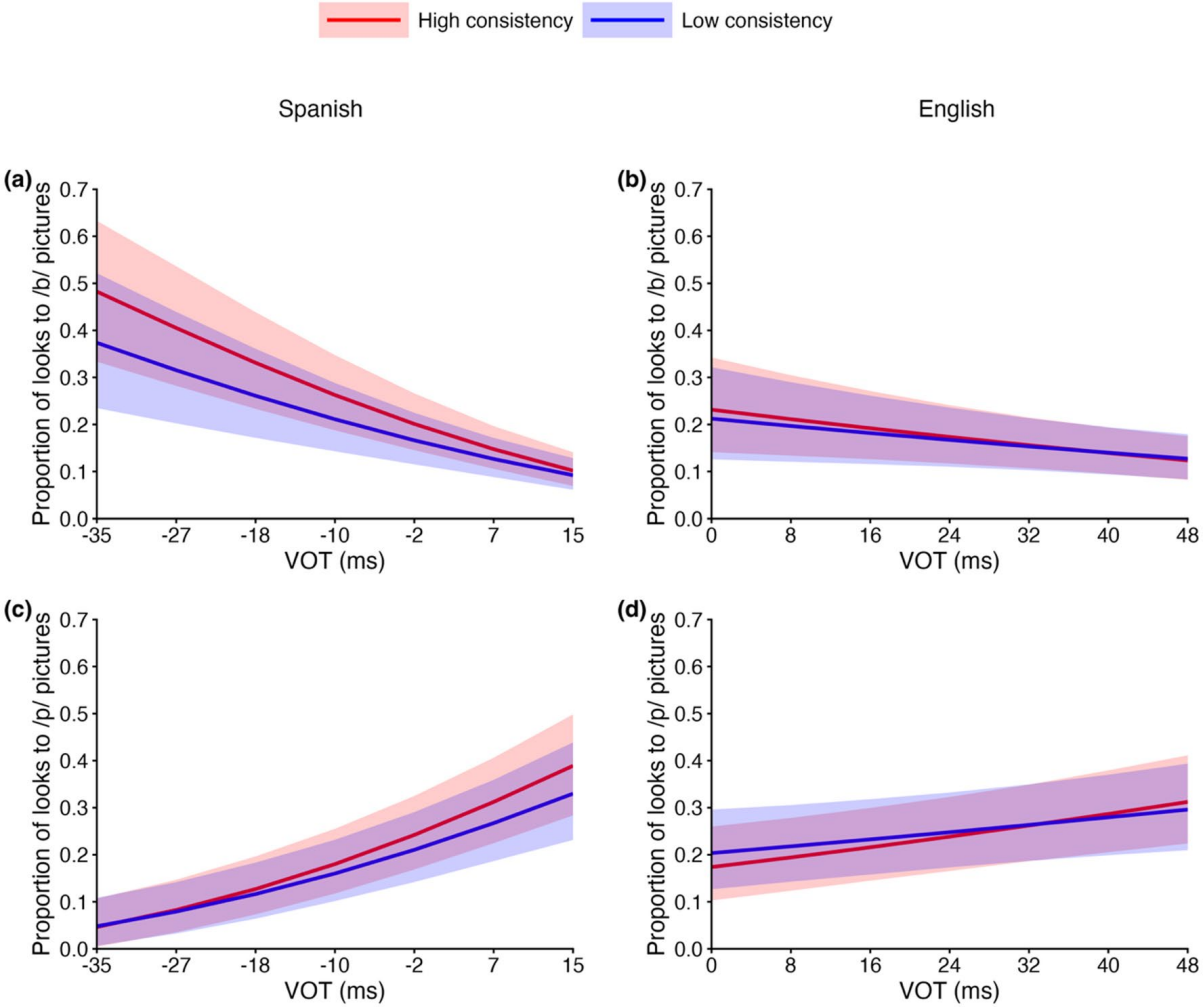
**(a**,** b)** Proportion of looks to /b/-onset pictures and **(c**,** d)** proportion of looks to /p/-onset pictures as a function of VOT and consistency for Spanish (left column) and English (right column). Lines represent model-predicted values. “Low consistency” represents one standard deviation below the mean, and “high consistency” represents one standard deviation above the mean. These values are shown for illustrative purposes only, as consistency was modeled as a continuous predictor. Values on both axes were back-transformed from the model’s transformed scale to their original scale for ease of interpretation. Shaded ribbons indicate 95% credible intervals around the model-predicted values.

Figure 5 shows the model-predicted proportion of looks to /b/-onset or/p/-onset pictures as a function of VOT and consistency for both Spanish (*N* = 65) and English (*N* = 52). First, we report the analyses of the proportion of looks to /b/-onset pictures in Spanish. As expected, there was decisive evidence for a main effect of VOT step, *β* = −0.24, 95% *CrI* (credible interval) [− 0.31, − 0.17], *BF* (Bayes Factor) = ∞ (Frequentist statistics are reported in Supplementary Information III), reflecting fewer looks to /b/-onset pictures as VOT increased overall. We also found moderate evidence for a main effect of consistency, *β* = 0.02, 95% *CrI* [− 0.01, 0.06], *BF* = 6.14, indicating that listeners with higher consistency showed a greater overall tendency to look at /b/-onset pictures. If more consistent listeners exhibit stronger initial lexical activation, we would expect an interaction between VOT step and consistency, such that listeners with higher consistency show more looks to /b/-onset pictures at lower VOT values than listeners with lower consistency. Indeed, we found strong evidence for this interaction, *β* = −0.01, 95% *CrI* [− 0.02, 0.00], *BF* = 15.63, indicating that listeners with higher consistency looked more to/b/-onset pictures when VOT was low (Fig. 5a). Complete results are presented in Supplementary Table S1.

Next, we report the analyses of the proportion of looks to /b/-onset pictures in English. There was very strong evidence for a main effect of VOT step, *β* = −0.08, 95% *CrI* [− 0.14, − 0.02], *BF* = 77.43, reflecting fewer looks to /b/-onset pictures as VOT increased overall. However, in contrast to the corresponding Spanish results, evidence for the interaction between VOT step and consistency was inconclusive, *β* = 0.00, 95% *CrI* [− 0.01, 0.00], *BF* = 1.32, indicating that the data did not provide clear support for either the presence or absence of an interaction (Fig. 5b). Evidence for a main effect of consistency was negligible. Complete results are presented in Supplementary Table S2.

For the proportion of looks to /p/-onset pictures in Spanish, there was decisive evidence for a main effect of VOT step, *β* = 0.26, 95% *CrI* [0.19, 0.33], *BF* = ∞, reflecting that listeners overall looked more to /p/-onset pictures as VOT increased. Although there was weak evidence for a main effect of consistency, *β* = 0.02, 95% *CrI* [− 0.01, 0.05], *BF* = 3.72, this effect was qualified by an interaction between VOT step and consistency, *β* = 0.01, 95% *CrI* [0.00, 0.01], *BF* = 4.42, with moderate evidence. This interaction indicates that listeners with higher consistency showed relatively more looks to /p/-onset pictures at higher VOT values, compared to listeners with lower consistency (Fig. 5c), mirroring the pattern observed for looks to /b/-onset pictures in Spanish (see Supplementary Table S3 for complete results).

Turning to /p/-onset pictures in English, as expected, there was again decisive evidence for a main effect of VOT step, *β* = 0.08, 95% *CrI* [0.03, 0.14], *BF* = 172.91, reflecting an overall increase in looks to /p/-onset pictures as VOT increased. Importantly, there was moderate evidence for an interaction between VOT step and consistency, *β* = 0.00, 95% *CrI* [0.00, 0.01], *BF* = 8.21. This pattern indicates that listeners with higher consistency showed relatively more looks to /p/-onset pictures at higher VOT values than listeners with lower consistency (Fig. 5d). Evidence for a main effect of consistency was negligible (see Supplementary Table S4 for complete results).

To sum up, speech perception consistency was associated with higher VOT-compatible initial lexical activation in most conditions. This effect was observed for both languages and both phoneme categories, with the exception of English /b/, which is likely more challenging due to its VOT overlap with Spanish /p/.

### Does consistency predict speech perception flexibility?

Our second research question examined whether consistency predicts speech perception flexibility. Speech perception flexibility was assessed using two measures: (1) recovery rate, defined as the proportion of garden-path trials in which participants ultimately fixated on the target after the point of disambiguation (POD), and (2) recovery latency, defined as the time taken to shift gaze to the target following the POD. Analyses were limited to garden-path trials, that is, trials in which participants initially fixated on a competitor (e.g., *peachpit* when hearing *peachball*) prior to the POD, because this fixation is a prerequisite for recovery. Here, we focus on acoustic distance from the target (hereafter *tDist*), consistency, and their interaction.

In the VWP, higher tDist corresponded to speech onsets that were more acoustically distant from the target (e.g., hearing the onset of *peachball*) and were therefore more misleading early in the signal. This increased the likelihood of a garden path (e.g., fixating *peachpit*) before the POD. After the POD (i.e., the point between *peach* and *ball*), listeners were expected to recover by shifting gaze to the target (e.g., *beachball*). Accordingly, higher recovery rates and shorter recovery latencies were taken to indicate greater speech perception flexibility.

**Fig. 6:**
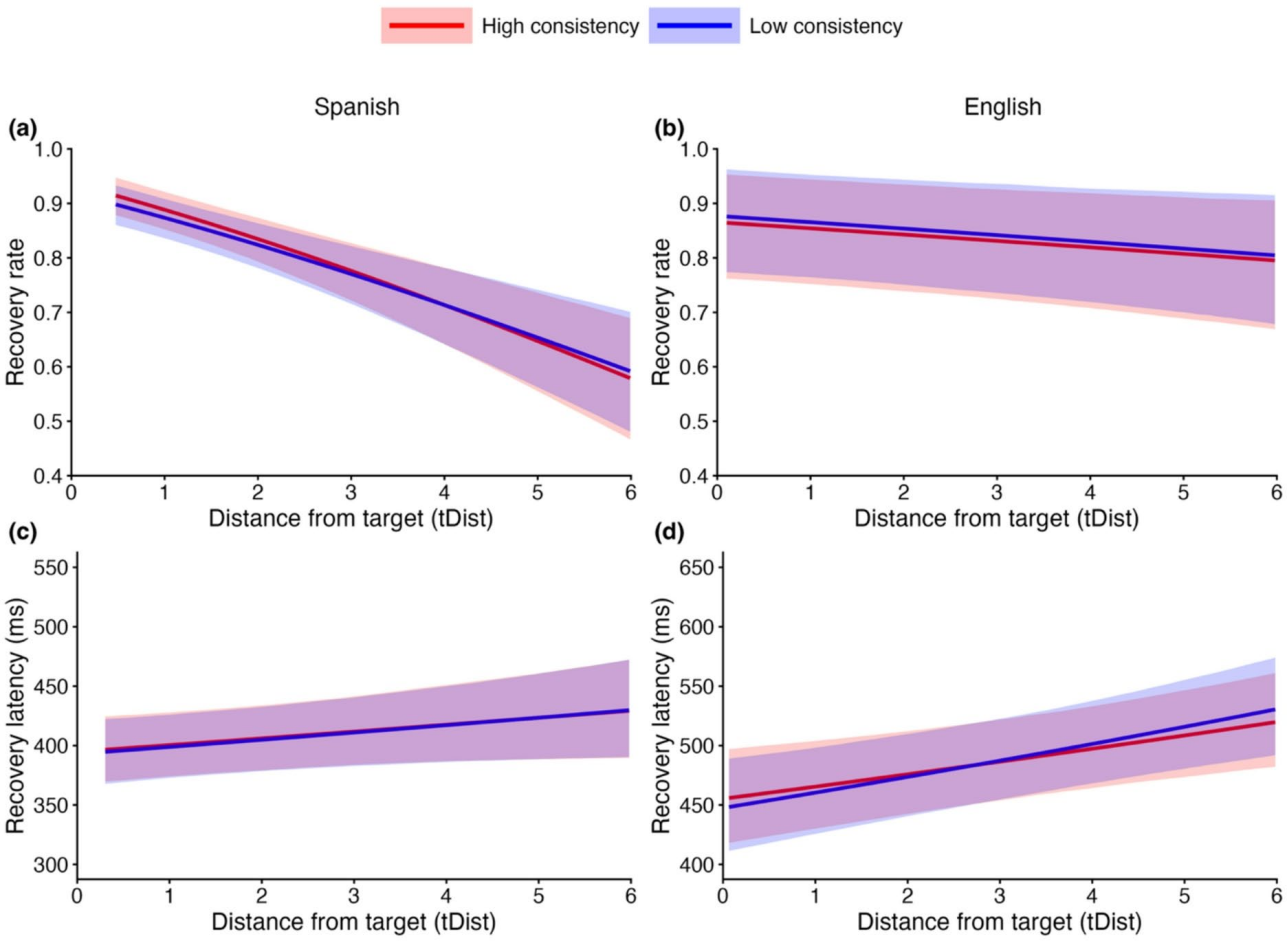
**(a**,** b)** Recovery rate and **(c**,** d)** recovery latency as a function of tDist and consistency for Spanish (left column) and English (right column). Lines represent model-predicted values. “Low consistency” represents one standard deviation below the mean, and “high consistency” represents one standard deviation above the mean. These values are shown for illustrative purposes only, as consistency was modeled as a continuous predictor. Values on both axes were back-transformed from the model’s transformed scale to their original scale for ease of interpretation. Shaded ribbons indicate 95% credible intervals around the model-predicted values.

Figure 6 shows the model-predicted recovery rate and recovery latency as a function of tDist and consistency for both Spanish (*N* = 65) and English (*N* = 52). As expected, as tDist increased, listeners were less likely to recover from a garden-path and took longer to do so in both Spanish and English, corresponding to strong to decisive evidence for a main effect of tDist across all four models (*BF* = 26.21–∞). Notably, evidence for both the main effect of consistency and the interaction between tDist and consistency ranged from inconclusive to strong evidence in favor of the null hypothesis (*BF* = 0.05–1.21). These results indicate that while acoustic distance influences recovery, speech perception consistency does not appear to enhance speech perception flexibility. Complete statistics are provided in Supplementary Tables S5–S8.

### Exploratory analyses: the role of L2 proficiency

To explore the possibility that L2 proficiency may strengthen cue-to-category mapping stability, thereby leading to greater speech perception consistency, we examined the relationship between L2 consistency and L2 proficiency (*N* = 56). L2 proficiency was measured using an English picture-naming task (see Methods for details). The correlation between L2 consistency and L2 proficiency was inconclusive, *ρ* = 0.21, *BF* = 1.14. This result provides no clear evidence for a relationship between L2 proficiency and L2 consistency.

We further explored whether L2 proficiency modulated the relationship between consistency and initial lexical activation by repeating the main analyses described above while including L2 proficiency as an additional predictor, testing both its main effect and its interactions with VOT step and consistency. Complete findings related to English proficiency for looks to pictures with /b/-onset and/p/-onset labels are reported in Supplementary Tables S9 and S10, respectively. In both models, the two-way interactions between VOT step and L2 proficiency were supported by decisive evidence, suggesting that listeners with higher L2 proficiency decreased looks to pictures of /b/-onset words more across the VOT continuum and increased looks to pictures of /p/-onset words more across the VOT continuum. However, the three-way interaction between VOT step, consistency, and L2 proficiency showed strong evidence for the null for /b/-onset items (*BF* = 0.05) and weak evidence for the null for /p/-onset items (*BF* = 0.60), suggesting that L2 proficiency does not modulate the relationship between consistency and initial lexical activation.

## Discussion

In this study, we investigated whether speech perception consistency influences initial lexical activation and/or speech perception flexibility in both L1 Spanish and L2 English. Our main finding was that consistency facilitated input-compatible initial lexical activation in most cases, but did not affect speech perception flexibility. Specifically, the results suggest that listeners with higher consistency show greater cue-to-category mapping stability, resulting in more early activation of acoustically compatible words in Spanish, and in English specifically for /p/-onset but not for /b/-onset words.

Previous work suggests that higher speech perception consistency is associated with more effective learning of unfamiliar phonetic contrasts^14,15^ and with better reading and language abilities^12,16,17^. The present study highlights another potential advantage of consistency: its role in facilitating initial lexical activation. Specifically, we found that more consistent listeners fixated on VOT-compatible pictures more frequently before the POD. Importantly, this effect was observed in three out of four tested cases: for both phonemes in L1 Spanish and for one phoneme (i.e., /p/) in L2 English, providing evidence for a role of consistency in early spoken-word recognition.

Consistent with the rationale outlined in our hypothesis, we interpret this effect as follows: the speech perception system of listeners with lower consistency is likely characterized by lower stability in cue-to-category mapping, resulting in increased uncertainty. Such uncertainty may lead listeners to adopt a “wait-and-see” strategy during the initial stages of spoken-word recognition; that is, to delay lexical activation to avoid premature commitment until more information becomes available^32^. Alternatively, listeners with lower consistency may activate lexical candidates in a less precise or more diffuse manner, resulting in weaker or noisier activation and more sustained competition among candidates. From this perspective, consistency may modulate the quality or selectivity of early lexical activation rather than its timing per se. The present data cannot fully distinguish between delayed activation and reduced selectivity, particularly given that the analyses focus on the early portion of the word before the point of disambiguation. Future studies are needed to disentangle these possibilities.

The current findings converge with results reported by a recent study^34^. The researchers asked English-speaking participants to complete VAS tasks with different continua including stop consonants (e.g., *beach–peach*, *dime–time*) and a VWP task with minimal pairs involving the same phonetic contrast (e.g., *bin–pin*). They found that listeners with higher speech perception consistency exhibited faster spoken-word recognition for stop consonants (but not for fricatives). Our proposed accounts may offer some insight into this pattern.

Importantly, our interpretations assume cue-to-category mapping as a potential source of consistency. However, the underlying sources of consistency remain unclear. Broadly, three possibilities can be considered across different levels of processing: (1) early sensory encoding, (2) mapping between acoustic cues and phoneme categories, and (3) downstream decision-level processes. As discussed below, our findings point most strongly to (2), the cue-to-category mapping level.

Consistency facilitated initial lexical activation but did not predict speech perception flexibility. This pattern makes a purely decision-level account less likely, because initial lexical activation reflects early processing before the point of disambiguation, occurring at around 450 ms. At the same time, consistency is unlikely to arise solely from early sensory encoding. Previous work has shown that consistency measured using the VAS task is not associated with the consistency of the frequency-following response (FFR^18^). This dissociation suggests that consistency does not primarily reflect variability in early acoustic encoding.

Taken together, the present findings are most consistent with the interpretation that consistency reflects the stability of the mapping between continuous acoustic input and phoneme categories. Under this account, listeners with higher consistency map acoustic cues onto phonological categories more reliably, which strengthens the influence of early acoustic evidence on lexical activation. In contrast, speech perception flexibility involves revising an initial interpretation once disambiguating information becomes available. This later process depends more on how listeners update lexical activation in response to new input than on how stable the initial cue-to-category mapping is. As a result, consistency may influence the strength of early lexical activation but not the ability to revise interpretations during speech perception.

Another factor to consider is that consistency may be modulated by top-down influences. This possibility is related to previous work linking consistency to language and reading abilities. For example, a previous study found that listeners were more consistent when processing words than nonwords, suggesting that lexical knowledge may contribute to stabilizing phonetic categorization through a feedback mechanism^35^. However, the present findings provide limited support for this interpretation. First, consistency predicted early lexical activation but not speech perception flexibility, even though flexibility plausibly places greater demands on lexical knowledge and top-down support than early activation. Second, if consistency were primarily driven by top-down feedback, listeners with higher L2 proficiency, who presumably possess stronger linguistic knowledge, might be expected to show higher consistency. However, we did not find clear evidence for a relationship between L2 consistency and L2 proficiency, and exploratory analyses further showed that proficiency did not interact with consistency in predicting initial lexical activation. These findings therefore do not support strong top-down effects on consistency.

Based on the arguments above, we suggest that speech perception consistency likely stems from the stability of cue-to-category mappings, with limited influence from top-down processes. Nonetheless, future neuroimaging studies are needed to further clarify the sources of consistency.

The inconclusive effect of consistency on initial lexical activation for English /b/ may reflect the fact that English /b/ is phonetically closer in VOT to Spanish /p/, making it particularly difficult for Spanish speakers to acquire. This pattern is consistent with the revised Speech Learning Model (SLM-r^36^), which predicts that L2 speech sounds that are phonetically similar to an existing L1 category are especially challenging to learn. Consequently, the absence of a consistency effect for English /b/ may reflect the greater difficulty associated with this sound, such that any facilitative role of consistency has not yet fully emerged. Moreover, while the effect of consistency is largely language-general, if we adopt the view that consistency reflects the stability of cue-to-category mapping, it is reasonable to expect that its functional impact would depend on how well a phonological contrast or category is established within a given language system. Consistent with this interpretation, we did not observe this effect for English /b/, and the effect size for English /p/ was smaller than that observed in L1 Spanish.

The weak relationship between consistency and speech perception flexibility, together with prior evidence that gradiency predicts flexibility^23,37^, suggests that consistency and gradiency serve distinct functions in speech processing. In the present paradigm, flexibility (indexed by recovery rate and recovery latency) reflects listeners’ ability to revise an initial misinterpretation of the input once disambiguating information becomes available. It is plausible that recovery from misleading input relies less on stable category commitments and more on listeners’ sensitivity to fine-grained acoustic variation and their ability to maintain multiple lexical candidates during processing^23,37^. These processes are more naturally captured by gradiency than by consistency. In contrast, consistency reflects the stability of phonetic categorization across repeated stimuli and may primarily support early, input-compatible lexical activation rather than late-stage revision. Consequently, lower consistency does not necessarily hinder recovery from misleading input: listeners with less stable categorization may initially activate lexical representations less strongly, but this weaker activation neither precludes nor facilitates flexible adjustment when disambiguating information becomes available. Taken together, these findings suggest that initial lexical activation and speech perception flexibility rely on dissociable mechanisms.

Recall that we found a positive correlation between L1 and L2 consistency. This finding suggests that consistency reflects an individual trait, aligning with previous evidence demonstrating positive correlations in consistency across tasks^15^, across phonetic categories^14,38^, and across modalities (visual vs. auditory^12^). Importantly, a previous study examining five vowels, two stops, and one fricative reported a stronger average correlation for consistency (*r* =.67) than for gradiency (*r* =.36)^38^. The present study extends these findings by demonstrating cross-language consistency in speech perception when listeners process the same acoustic cue across languages. In contrast, gradiency did not show cross-language alignment^23^. Taken together, these findings are consistent with the possibility that consistency reflects a more general mechanism than gradiency, although broader conclusions would require evidence across additional contrasts and languages.

Speech perception consistency appears to align between L1 and L2 overall, including among lower-proficiency listeners. In contrast, gradiency showed little convergence across languages, with only weak evidence for a cross-language association; however, gradiency was positively correlated across languages among higher-proficiency participants, suggesting that gradiency may become more stable with increased language experience^23^. One possible (albeit speculative) interpretation is that L2 consistency may develop earlier than L2 gradiency within the same individuals. Consistency likely depends on accumulating sufficient input to establish stable cue-to-category mappings. Once these mappings are stable, finer gradations within categories can begin to influence perception, giving rise to gradiency. In this view, consistency provides the foundational stability upon which gradiency operates, supporting flexible and efficient speech perception. Consistent with this proposal, recent longitudinal work tracking school-aged children appears to indicate that consistency improves over the first several years of development, whereas gradiency appears to unfold over a more extended time span^39^. Future longitudinal research in adults will be essential to test this proposed developmental sequence directly.

## Conclusion

We found that speech perception consistency facilitates initial lexical activation across L1 Spanish and L2 English (for /p/-onset words only). Listeners with higher consistency tended to look at the VOT-compatible pictures more than those with lower consistency at the early moments of hearing a word. This pattern suggests that listeners with lower consistency may delay lexical activation or activate lexical candidates less selectively during the early stages of spoken-word recognition. While our findings point to cue-to-category mapping stability as a likely source of speech perception consistency, further research is needed to confirm this interpretation. Unraveling the sources of consistency is particularly important given the multiple advantages that consistency appears to confer across speech and language processing.

## Methods

Participants, tasks, and procedures were reported in^23^. Here, we summarize the key aspects relevant to the present analyses.

### Participants

Participants included 70 L1 Spanish speakers (53 females) residing in San Sebastian, Spain, who varied in their English proficiency. They were between 18 and 40 years of age (*M* = 27.8) and had normal or corrected-to-normal vision, with no reported hearing or neurological impairments. The study was approved by the Basque Center on Cognition, Brain and Language (BCBL) Ethics Review Board (ref. 030423SM) and adhered to the principles of the Declaration of Helsinki. All participants provided written informed consent and received monetary compensation for their participation.

### Tasks

#### VAS

Participants categorized stimuli drawn from a natural-speech “buh”–“puh” continuum in English and a “ba”–“pa” continuum in Spanish, with each continuum varying factorially in VOT and fundamental frequency (F_0_; the acoustic correlate of pitch). In both languages, the stimuli were recorded by a speaker of the corresponding language. The continua comprised seven VOT steps (Spanish: −35 to + 10 ms; English: 1 to 45 ms; step size ≈ 7–8 ms) and five F_0_ steps (Spanish: 179–193 Hz in 3–4 Hz increments; English: 90–125 Hz in 8.75 Hz increments). For Spanish, the VOT continuum was generated using a VOT-synthesis script^40^, which allows precise manipulation of both VOT and F_0_. For English, to ensure comparability with the original study, the stimuli were taken from^11^, in which the VOT continuum was created via progressive cross-splicing^41^. At each VOT step, the pitch contour was extracted and manipulated using the pitch-synchronous overlap-add (PSOLA) algorithm in Praat to generate the F_0_ variation (see^11^ for details).

Participants completed the VAS task in both English and Spanish and were informed in advance which language would be used in each block. On every trial, a horizontal line labeled at its endpoints with the two relevant speech categories was displayed (English: *buh*–*puh*; Spanish: *ba*–*pa*), with category positions held constant across trials. After hearing each stimulus, participants indicated their percept by clicking anywhere along the line. A rectangular bar then appeared at the selected location, and participants could adjust their response or confirm it by pressing the space bar. Stimuli were presented in a randomized order within each language. Each item was repeated three times, yielding 105 trials per language.

### VWP

For both Spanish and English, participants heard word pairs that differed only in their initial voicing (/b/ vs. /p/). Each pair was manipulated along a seven-step VOT continuum to create word and nonword variants (e.g., *beachball–peachball*). Ambiguity at the word onset was resolved by disambiguating information at the word offset (e.g., “–ball” vs. “–pit”). This design allowed us to assess initial lexical activation (i.e., the proportion of fixations to /b/- and /p/-onset pictures across VOT) and speech perception flexibility (i.e., the rate and speed of recovery from misleading speech input).

For the Spanish stimuli, we created a set of 20 words, including 10 critical items forming five /b/–/p/ minimal pairs (e.g., *balanza [scale]–palacio [palace]*) and five filler pairs contrasting /l/ and /r/ onsets (e.g., *lavabo [sink]–regalo [gift])*. Recordings were made by a Spanish speaker in a sound-attenuated room. The Spanish stimuli closely matched the English stimuli in length, syllable structure, and phonemic overlap. The English stimuli were identical to those used in^37^.

Seven-step VOT continua were created for each /b/–/p/ pair (Spanish: −35 to + 15 ms; English: 0 to + 48 ms). As with the VAS stimuli, Spanish stimuli were manipulated using the VOT-synthesis script^40^ while English stimuli were taken from^37^. This yields 140 experimental items. Each item was presented three times, resulting in 420 experimental trials. An equal number of filler trials using correctly produced and misarticulated /l/–/r/ items were interspersed, bringing the total to 840 trials. Experimental and filler items were grouped into five four-item sets per language (e.g., *balanza*, *palacio*, *lavabo*, and *regalo* formed one set in the Spanish VWP), each containing four semantically unrelated words with matched stress patterns. Images corresponding to each word were primarily sourced from the MultiPic database^42^.

Participants first completed a familiarization phase in which each image was presented individually together with its corresponding label. This was followed by eye-tracker calibration and instructions for the task. At the start of each trial in the main experiment, a red circle appeared at the center of the screen, accompanied by four pictures and an “X” arranged in a pentagonal layout. After 500 ms, the circle turned blue, prompting participants to click on it to hear a word. Participants then selected the corresponding image or clicked the “X” if none of the images matched the spoken word.

Both the VAS and the VWP were programmed in Experiment Builder (version 2022.2.5; SR Research Ltd., 2022). For the VWP, eye movements were recorded with an EyeLink 1000 Plus system (SR Research Ltd.) at a 1,000 Hz sampling rate in a head-stabilized setup.

### English picture naming task

To assess English proficiency, we administered an English picture-naming task^43^. The task consisted of 65 images corresponding to noncognate words selected from the MultiPic database^42^. On each trial, participants viewed a picture and typed a single English word to name the object shown. The stimuli covered a range of semantic categories, including animals (24 items) and body parts (eight items). Participants received a score between 0 and 65 based on the total number of correctly named pictures. The task was programmed using jsPsych (Version 7.3.1^44^) and administered through the Chrome browser.

### Procedure

Participants were randomly assigned to two counterbalanced groups. Each participant attended two lab sessions held seven to fourteen days apart (approximately 1.5 h and 1 h, respectively). Half of the participants completed the Spanish VAS and VWP tasks in the first session, whereas the other half completed the English versions first. Prior to the English session, participants completed an online questionnaire (measuring language background and musical training) and a short training task designed to familiarize them with the English stimuli. The English picture-naming task always took place in the first lab session. In addition to the three main tasks, participants completed a spatial Stroop task (assessing inhibitory control) and a Corsi block-tapping task (assessing working memory) in the first lab session. These additional tasks are not discussed further, as they are not of theoretical interest here and did not improve model fit.

### Data preprocessing

#### VAS: consistency

Click locations along the x-axis were converted from pixel values to VAS ratings ranging from 0 to 100. Curve fitting was performed using a four-parameter rotated logistic function to each participant’s responses in MATLAB (version R2022a; The MathWorks Inc., Natick, MA, USA), using a constrained least-squares procedure (code from^45^). Consistency was quantified by first computing trial-wise residuals, defined as the difference between the observed VAS rating on each trial and the value predicted by the participant’s fitted curve for that stimulus. The standard deviation of these residuals was then calculated for each participant, with larger values indicating lower response consistency. To facilitate interpretation, this measure was multiplied by −1, such that higher values reflect greater consistency.

No participants were excluded from the Spanish VAS. In the English VAS, 14 participants were excluded due to a restricted response range (i.e., the difference between the mean ratings at Step 7 and Step 1 was less than 25 units). This narrow range resulted in poor model fits and unreliable estimates of consistency, and these participants were therefore excluded from the English analyses. Importantly, these 14 participants provided typical response ranges in the Spanish VAS, suggesting reduced robustness of bilabial category representations in English rather than inattentiveness. Accordingly, these participants were excluded only from analyses involving English consistency; all participants were retained for the Spanish analyses. The remaining model fits were satisfactory, with mean *R*^2^ values of 0.940 for Spanish and 0.893 for English.

### VWP: eye-tracking data

The eye-tracking data, recorded from the onset of the trial (the appearance of the blue circle) to the participant’s response, were automatically parsed into saccades and fixations using default EyeLink psychophysical parameters. Data preprocessing was conducted using EyelinkAnalysis (Version 5.41^46^). Adjacent saccades and fixations were merged into a single look, commencing at the onset of the saccade and concluding at the fixation offset. For analyses, the eye-tracking data were downsampled to 250 Hz, and a fixed trial duration of 2,000 ms relative to stimulus onset was established. In instances where trials terminated before this point, the last eye movement was extended. Conversely, trials surpassing the 2,000-ms threshold were truncated. Data from five participants were excluded from the Spanish VWP and data from four participants were excluded from the English VWP because they fixated on the selected picture in fewer than 60% of trials during the 300-ms window preceding the click; this pattern either meant that they did not reliably fixate the selected picture at the time of the response or that the eye-tracker failed to record reliable gaze data. As in the VAS, participant exclusions were applied on a task-specific basis. Therefore, exclusion from one task did not result in exclusion from the other tasks.

### Statistical Analyses

We operationalized initial lexical activation as trials in which participants fixated on the /b/- and/p/-onset pictures across the VOT continuum before the point of disambiguation (POD). The POD refers to the moment in the auditory signal at which the input uniquely identifies the target word (e.g., the time point between *beach* and *ball* in *beachball*). Speech perception flexibility was measured using two indices: (1) recovery rate, defined as the proportion of garden-path trials in which participants ultimately fixated on the target after the POD, and (2) recovery latency, defined as the time required to shift gaze to the target after the POD. All measurements were corrected for a 200-ms oculomotor delay^47,48^.

We fitted eight Bayesian linear mixed-effects models using brms^49^in R (version 4.3.2^50^) via RStudio (version 2023.12.0 + 369^51^) to examine whether speech perception consistency predicted initial lexical activation, recovery rate, and recovery latency in Spanish and English. Graphs were created with ggplot2 package^52^.

For initial lexical activation, we fitted two models for each language: one with the proportion of looks to /b/-onset pictures as the dependent variable and one with the proportion of looks to /p/-onset pictures as the dependent variable. Only looks occurring before the POD were included in the analyses. Each model included VOT step (centered) and consistency (centered) as predictors. Proportions of looks were transformed using an empirical logit (i.e., the number of trials with looks to /b/-onset or /p/-onset pictures divided by the total of six trials per condition cell). The model included by-subject and by-item random intercepts and random slopes for VOT step. Higher proportions of looks to /b/-onset pictures at low VOT values and to /p/-onset pictures at high VOT values, as indexed by an interaction between VOT step and consistency, were interpreted as stronger VOT-compatible initial lexical activation.

To examine speech perception flexibility, the VOT step of the initial phoneme was recoded as its acoustic distance from the target (hereafter *tDist*), ranging from 0 to 6. For instance, a stimulus at VOT Step 1 (− 35 ms in Spanish; 0 ms in English) was assigned tDist = 0 when the target was an extreme /b/ item (e.g., *balanza*, *beachball*) and tDist = 6 when the target was an extreme /p/ item (e.g., *balacio*, *beachpit*). Each model included tDist (centered) and consistency (centered) as predictors. Recovery rate and recovery latency were analyzed in separate models for each language. Analyses were restricted to garden-path trials, that is, trials in which participants initially fixated on a competitor (e.g., *peachpit* when hearing ϸeachball) before the POD, as this fixation is a prerequisite for recovery. The model included by-subject and by-item random intercepts and random slopes for tDist. Higher recovery rates and shorter recovery latencies indicate greater speech perception flexibility.

We used Bayes Factors (BFs) to quantify the strength of evidence for or against specific effects. We interpreted BFs following^53^: values between 1 and 3 indicate weak evidence, 3–10 moderate, 10–30 strong, 30–100 very strong, and > 100 decisive evidence in favor of an effect; conversely, values between 0.33 and 1 indicate weak evidence, 0.1–0.33 moderate, 0.03–0.1 strong, 0.01–0.03 very strong, and < 0.01 decisive evidence against an effect. Values close to 1 indicate inconclusive evidence. Effects were considered meaningful when the BF indicated at least moderate evidence in favor of an effect.

We also conducted frequentist analyses using the same model structure and predictors as in the Bayesian analyses to ensure that the effects were robust across analytical approaches. The results are reported in Supplementary Tables S11–18 and showed the same pattern of findings.

## Supplementary Information

Below is the link to the electronic supplementary material.

Supplementary Material 1

## Data Availability

The datasets generated during and/or analyzed during the current study are available in the OSF repository, [https://doi.org/10.17605/OSF.IO/QXMYK](https:/doi.org/10.17605/OSF.IO/QXMYK).
