# Supplementary material for: Speech perception consistency facilitates initial lexical activation, but not speech perception flexibility
Source: Sci Rep. 2026 Apr 9;16:16189. doi: 10.1038/s41598-026-47943-3 (PMC13201790; doi:10.1038/s41598-026-47943-3)
Supplement: Supplementary file 1 — Supplementary Material 1 [file 41598_2026_47943_MOESM1_ESM.docx]

**Speech Perception Consistency Facilitates Initial Lexical Activation, but Not Speech Perception Flexibility**

Brian W. L. Wong^a,b^, Arthur G. Samuel^a,c,d^, Efthymia C. Kapnoula^a,d^

^a^ BCBL, Basque Center on Cognition, Brain and Language, Donostia−San Sebastian, Spain

^b^ University of the Basque Country (UPV−EHU), Spain

^c^ Department of Psychology, Stony Brook University, New York, U.S.A.

^d^ Ikerbasque, Basque Foundation for Science, Bilbao, Spain

**ONLINE SUPPLEMENTARY INFORMATION**

**Supplementary Information I: Statistical Tables for the Main Analyses**

|  | Estimate | Est. Error | 95% *CrI* | *BF* |
| --- | --- | --- | --- | --- |
| (Intercept) | −1.22 | 0.13 | [−1.48, −0.96] | — |
| VOTstep | −0.24 | 0.04 | [−0.31, −0.17] | Inf |
| Con | 0.02 | 0.02 | [−0.01, 0.06] | 6.14 |
| VOTstep × Con | −0.01 | 0.00 | [−0.02, −0.00] | 15.63 |

**Table S1.** Bayesian model estimates for the proportion of looks to /b/-onset pictures and consistency for Spanish. Note. Est. error = standard error of the estimate; 95% CrI = 95% credible interval; BF = Bayes Factor; Inf = infinite; VOT = voice onset time; Con = consistency.

|  | Estimate | Est. Error | 95% *CrI* | *BF* |
| --- | --- | --- | --- | --- |
| (Intercept) | −1.36 | 0.13 | [−1.62, −1.10] | — |
| VOTstep | −0.08 | 0.03 | [−0.14, −0.02] | 77.43 |
| Con | 0.00 | 0.01 | [−0.02, 0.02] | 0.10 |
| VOTstep × Con | 0.00 | 0.00 | [−0.01, 0.00] | 1.32 |

**Table S2.** Bayesian model estimates for the proportion of looks to /b/-onset pictures and consistency for English. Note. Est. error = standard error of the estimate; 95% CrI = 95% credible interval; BF = Bayes Factor; VOT = voice onset time; Con = consistency.

|  | Estimate | Est. Error | 95% *CrI* | *BF* |
| --- | --- | --- | --- | --- |
| (Intercept) | −1.02 | 0.16 | [−1.34, −0.70] | — |
| VOTstep | 0.26 | 0.04 | [0.19, 0.33] | Inf |
| Con | 0.02 | 0.02 | [−0.01, 0.05] | 3.72 |
| VOTstep × Con | 0.01 | 0.00 | [0.00, 0.01] | 4.42 |

**Table S3.** Bayesian model estimates for the proportion of looks to /p/-onset pictures and consistency for Spanish. Note. Est. error = standard error of the estimate; 95% CrI = 95% credible interval; BF = Bayes Factor; Inf = infinite; VOT = voice onset time; Con = consistency.

|  | Estimate | Est. Error | 95% *CrI* | *BF* |
| --- | --- | --- | --- | --- |
| (Intercept) | −0.86 | 0.15 | [−1.15, −0.57] | — |
| VOTstep | 0.08 | 0.03 | [0.03, 0.14] | 172.91 |
| Con | 0.00 | 0.01 | [−0.03, 0.03] | 0.03 |
| VOTstep × Con | 0.00 | 0.00 | [0.00, 0.01] | 8.21 |

**Table S4.** Bayesian model estimates for the proportion of looks to /p/-onset pictures and consistency for English. Note. Est. error = standard error of the estimate; 95% CrI = 95% credible interval; BF = Bayes Factor; VOT = voice onset time; Con = consistency.

|  | Estimate | Est. Error | 95% *CrI* | *BF* |
| --- | --- | --- | --- | --- |
| (Intercept) | 0.68 | 0.08 | [0.53, 0.83] | — |
| tDist | −0.18 | 0.02 | [−0.23, −0.13] | Inf |
| Con | 0.00 | 0.02 | [−0.03, 0.03] | 0.07 |
| tDist × Con | 0.00 | 0.01 | [−0.01, 0.01] | 0.62 |

**Table S5.** Bayesian model estimates for recovery rate and consistency for Spanish. Note. Est. error = standard error of the estimate; 95% CrI = 95% credible interval; BF = Bayes Factor; tDist = distance from the target; Con = consistency; Inf = infinite.

|  | Estimate | Est. Error | 95% *CrI* | *BF* |
| --- | --- | --- | --- | --- |
| (Intercept) | 0.97 | 0.16 | [0.65, 1.30] | — |
| tDist | −0.04 | 0.02 | [−0.08, −0.01] | 34.09 |
| Con | 0.00 | 0.01 | [−0.02, 0.01] | 0.35 |
| tDist × Con | 0.00 | 0.00 | [0.00, 0.00] | 0.08 |

**Table S6.** Bayesian model estimates for recovery rate and consistency for English. Note. Est. error = standard error of the estimate; 95% CrI = 95% credible interval; BF = Bayes Factor; tDist = distance from the target; Con = consistency.

|  | Estimate | Est. Error | 95% *CrI* | *BF* |
| --- | --- | --- | --- | --- |
| (Intercept) | 6.02 | 0.03 | [5.96, 6.09] | — |
| tDist | 0.01 | 0.01 | [0.00, 0.03] | 26.21 |
| Con | 0.00 | 0.00 | [−0.01, 0.01] | 0.05 |
| tDist × Con | 0.00 | 0.00 | [0.00, 0.00] | 0.10 |

**Table S7.** Bayesian model estimates for recovery latency and consistency for Spanish. Note. Est. error = standard error of the estimate; 95% CrI = 95% credible interval; BF = Bayes Factor; tDist = distance from the target; Con = consistency.

|  | Estimate | Est. Error | *95% CrI* | *BF* |
| --- | --- | --- | --- | --- |
| (Intercept) | 6.19 | 0.03 | [6.13, 6.25] | — |
| tDist | 0.03 | 0.01 | [0.01, 0.04] | 1,332.33 |
| Con | 0.00 | 0.00 | [−0.01, 0.00] | 0.05 |
| tDist × Con | 0.00 | 0.00 | [0.00, 0.00] | 1.21 |

**Table S8.** Bayesian model estimates for recovery latency and consistency for English. Note. Est. error = standard error of the estimate; 95% CrI = 95% credible interval; BF = Bayes Factor; tDist = distance from the target; Con = consistency.

**Supplementary Information II: Statistical Tables for the Exploratory Analyses**

|  | Estimate | Est. Error | 95% *CrI* | *BF* |
| --- | --- | --- | --- | --- |
| (Intercept) | −1.35 | 0.13 | [−1.61, −1.09] | — |
| English proficiency | −0.01 | 0.01 | [−0.03, 0.00] | 5.21 |
| VOTstep × English proficiency | −0.01 | 0.00 | [−0.01, 0.00] | 2,665.67 |
| Con × English proficiency | 0.00 | 0.00 | [0.00, 0.00] | 0.53 |
| VOTstep × Con × English proficiency | 0.00 | 0.00 | [0.00, 0.00] | 0.05 |

**Table S9.** Bayesian model estimates for the proportion of looks to /b/-onset pictures, consistency, and proficiency for English. Note. Est. error = standard error of the estimate; 95% CrI = 95% credible interval; BF = Bayes Factor; Inf = infinite; VOT = voice onset time; Con = consistency.

|  | Estimate | Est. Error | 95% *CrI* | *BF* |
| --- | --- | --- | --- | --- |
| (Intercept) | −0.85 | 0.15 | [−1.15, −0.57] | — |
| English proficiency | −0.02 | 0.01 | [−0.04, 0.00] | 17.91 |
| VOTstep × English proficiency | 0.01 | 0.00 | [0.00, 0.01] | 7,999 |
| Con × English proficiency | 0.00 | 0.00 | [0.00, 0.00] | 0.42 |
| VOTstep × Con × English proficiency | 0.00 | 0.00 | [0.00, 0.00] | 0.60 |

**Table S10.** Bayesian model estimates for the proportion of looks to /p/-onset pictures, consistency, and proficiency for English. Note. Est. error = standard error of the estimate; 95% CrI = 95% credible interval; BF = Bayes Factor; Inf = infinite; VOT = voice onset time; Con = consistency.

**Supplementary Information III: Frequentist Statistics for the Main Analyses**

|  | Estimate | *SE* | *df* | *t* | *p* |
| --- | --- | --- | --- | --- | --- |
| (Intercept) | −1.22 | 0.11 | 17.19 | −11.21 | .00 |
| VOTstep | −0.24 | 0.01 | 4433.51 | −36.37 | .00 |
| Con | 0.02 | 0.02 | 64.04 | 1.52 | .13 |
| VOTstep × Con | −0.01 | 0.00 | 4433.51 | −3.86 | .00 |

**Table S11.** Frequentist model estimates for the proportion of looks to /b/-onset pictures and consistency for Spanish. Note.  SE = standard error; df = degrees of freedom; VOT = voice onset time; Con = consistency.

|  | Estimate | *SE* | *df* | *t* | *p* |
| --- | --- | --- | --- | --- | --- |
| (Intercept) | −1.36 | 0.11 | 18.81 | −12.57 | .00 |
| VOTstep | −0.08 | 0.01 | 3508.64 | −11.11 | .00 |
| Con | 0.00 | 0.01 | 51.10 | 0.12 | .91 |
| VOTstep × Con | 0.00 | 0.00 | 3508.64 | −1.29 | .20 |

**Table S12.** Frequentist model estimates for the proportion of looks to /b/-onset pictures and consistency for English. Note.  SE = standard error; df = degrees of freedom; VOT = voice onset time; Con = consistency.

|  | Estimate | *SE* | *df* | *t* | *p* |
| --- | --- | --- | --- | --- | --- |
| (Intercept) | −1.02 | 0.13 | 14.11 | −7.79 | .00 |
| VOTstep | 0.27 | 0.01 | 4433.80 | 39.74 | .00 |
| Con | 0.02 | 0.02 | 64.17 | 1.31 | .19 |
| VOTstep × Con | 0.01 | 0.00 | 4433.80 | 2.67 | .01 |

**Table S13.** Frequentist model estimates for the proportion of looks to /p/-onset pictures and consistency for Spanish. Note.  SE = standard error; df = degrees of freedom; VOT = voice onset time; Con = consistency.

|  | Estimate | *SE* | *df* | *t* | *p* |
| --- | --- | --- | --- | --- | --- |
| (Intercept) | −0.86 | 0.12 | 26.35 | −6.94 | .00 |
| VOTstep | 0.08 | 0.01 | 3508.73 | 10.93 | .00 |
| Con | 0.00 | 0.01 | 51.44 | −0.02 | .98 |
| VOTstep × Con | 0.00 | 0.00 | 3508.73 | 2.26 | .02 |

**Table S14.** Frequentist model estimates for the proportion of looks to /p/-onset pictures and consistency for English. Note.  SE = standard error; df = degrees of freedom; VOT = voice onset time; Con = consistency.

|  | Estimate | *SE* | *df* | *t* | *p* |
| --- | --- | --- | --- | --- | --- |
| (Intercept) | 0.66 | 0.08 | 36.98 | 8.66 | .00 |
| tDist | −0.17 | 0.01 | 2943.35 | −18.47 | .00 |
| Con | 0.00 | 0.02 | 64.86 | 0.14 | .89 |
| tDist × Con | 0.00 | 0.00 | 2940.44 | −0.94 | .35 |

**Table S15.** Frequentist model estimates for recovery rate and consistency for Spanish. Note.  SE = standard error; df = degrees of freedom; tDist = distance from the target; Con = consistency.

|  | Estimate | *SE* | *df* | *t* | *p* |
| --- | --- | --- | --- | --- | --- |
| (Intercept) | 0.97 | 0.13 | 14.04 | 7.60 | .00 |
| tDist | −0.03 | 0.01 | 2491.19 | −3.85 | .00 |
| Con | 0.00 | 0.01 | 45.39 | −0.35 | .73 |
| tDist × Con | 0.00 | 0.00 | 2487.76 | 0.21 | .83 |

**Table S16.** Frequentist model estimates for recovery rate and consistency for English. Note.  SE = standard error; df = degrees of freedom; tDist = distance from the target; Con = consistency.

|  | Estimate | *SE* | *df* | *t* | *p* |
| --- | --- | --- | --- | --- | --- |
| (Intercept) | 6.02 | 0.03 | 12.91 | 210.89 | .00 |
| tDist | 0.02 | 0.00 | 2484.71 | 4.30 | .00 |
| Con | 0.00 | 0.00 | 64.96 | −0.01 | .99 |
| tDist × Con | 0.00 | 0.00 | 2489.14 | 0.03 | .98 |

**Table S17.** Frequentist model estimates for recovery latency and consistency for Spanish. Note.  SE = standard error; df = degrees of freedom; tDist = distance from the target; Con = consistency.

|  | Estimate | *SE* | *df* | *t* | *p* |
| --- | --- | --- | --- | --- | --- |
| (Intercept) | 6.19 | 0.03 | 17.19 | 241.47 | .00 |
| tDist | 0.03 | 0.00 | 2242.48 | 6.73 | .00 |
| Con | 0.00 | 0.00 | 45.75 | −0.06 | .95 |
| tDist × Con | 0.00 | 0.00 | 2238.44 | −0.88 | .38 |

**Table S18.** Frequentist model estimates for recovery latency and consistency for English. Note.  SE = standard error; df = degrees of freedom; tDist = distance from the target; Con = consistency.
